# Tubercle Bacillus

**Published:** 1886-12

**Authors:** 


THE TUBERCLE BACILLUS.

In quite a large number of cases of tuberculosis and chronic bronchitis the greater portion of the sputum is produced by the mucous membranes, and the Bacillus Tuberculosis, though present, either is not found, or is found only after a long search. In the *Berliner Klin. Wochenschrift* for October 18th, 1886, Biedert suggests the following method by which time is saved and greater certainty reached :

A dessert spoonful of the sputum is mixed with a dessert spoonful of water and 15 drops sodic hydrate. This is boiled till the sputum is all dissolved, when 4 dessert spoonfuls of water are added and the whole again boiled until a homogeneous fluid with only a few floating particles is obtained. If, when cold, the solution is not perfectly thin, 3 to 6 dessert spoonfuls of water are again added. The whole is then put into a conical glass and allowed to stand from two to three days. By this time the bacilli, if present, have fallen to the bottom of the glass. The supernatant fluid is carefully drawn off and the deposit in the bottom will contain all of the bacilli in the whole mass of sputum used. A small quantity of egg albumen may be added to the deposit for the purpose of fixing it to the cover glass during the process of coloring. When the bacillus is not found by the ordinary methods in suspected cases this procedure may help largely in reaching a correct diagnosis.

